# Can initial diagnostic PET-CT aid to localize tumor bed in breast cancer radiotherapy: feasibility study using deformable image registration

**DOI:** 10.1186/1748-717X-8-163

**Published:** 2013-07-03

**Authors:** Oyeon Cho, Mison Chun, Young-Taek Oh, Mi-Hwa Kim, Hae-Jin Park, Jae-Sung Heo, O Kyu Noh

**Affiliations:** 1 Department of Radiation Oncology, Ajou University School of Medicine, Suwon, Republic of Korea

**Keywords:** PET-CT, Tumor bed, Breast cancer, Radiotherapy, Deformable image registration

## Abstract

**Background:**

Localization of the tumor bed of breast cancer is crucial for accurate planning of boost irradiation. Lumpectomy cavity and surgical clips provide localizing information about tumor bed. However, defining the tumor bed is often difficult because of presence of unclear lumpectomy cavity and lack of certain information such as absence of surgical clips. In the present study, we evaluated the feasibility of initial diagnostic PET-CT in localization of the tumor bed using deformable image registration (DIR).

**Methods:**

We selected twenty-five patients who had an initial diagnostic PET-CT performed and underwent breast-conserving surgery with surgical clips in tumor bed. In every individual patient, two target volumes were separately delineated on planning CT; 1) target volume based on surgical clips with a margin of 1 cm (TV_clip_) and 2) tumor volume based on 90% of maximum SUV on PET-CT registered by DIR (TV_PET_). The percent of TV_PET_ in TV_clip_ (V_in_) was calculated and distance between center points of two volumes (D_center_) was also measured.

**Results:**

Mean D_center_ between two volumes was 1.4 cm (range, 0.33 – 2.53). Mean V_in_ was 94.8% (range, 60.9-100) and 100% in 18 out of 25 patients. When compared to the center of TV_clip_, the center of TV_PET_ tended to be located posteriorly (mean 0.3 cm, standard deviation 0.6), laterally (mean 0.3 cm, standard deviation 0.8) and inferiorly (mean 0.4 cm, standard deviation 0.9).

**Conclusion:**

Initial diagnostic PET-CT can be one of the possible references to localize the tumor bed in breast cancer radiotherapy.

## Introduction

Whole breast irradiation after breast conserving surgery is one of the standard treatments for the patients with early-stage breast cancer [1,2]. After the whole breast irradiation, additional boost to the tumor bed have demonstrated considerable improvement in local control rate [3,4]. For achieving the adequate local control of the boost irradiation, appropriate localization of the tumor bed is crucial. The accuracy of defining the tumor bed has been improved by the use of CT-based simulation, in which lumpectomy cavity and surgical clips provide localizing information [5-7]. However, because of the poor visualization of lumpectomy cavity and absence of surgical clips, it is difficult to determine the tumor bed in some cases. In such cases, radiation oncologists delineate the tumor bed referenced by surgical scar marking and preoperative imaging studies including mammography, ultrasonography, and breast MR. However, surgical scar considering good cosmetic outcome does not always represents the original location of tumor bed. Because the position at preoperative imaging is different from the planning CT, there may be a geographic miss in estimation of tumor bed. Some studies suggest that breast MR identically positioned with planning CT provides more precise information on tumor bed localization [6,8]. However, the additional imaging study for radiotherapy planning to localize the tumor bed is not always available.

Recently, the method of deformable image registration (DIR) has been developed and evaluated for the purpose of the more precise image-guided radiotherapy and adoptive radiotherapy [9-11]. Based on deformable image registration, we hypothesized that the localization of tumor bed may be feasible using preoperative imaging work-up without requirement of additional study for the sole purpose of radiotherapy planning. For deformable image registration with planning CT, we selected the diagnostic PET-CT as a reference imaging study. Although, preoperative CT imaging without PET can aid to delineate the boost volume with reduction of the inter-observer variation [12], preoperative PET-CT, the use of which has been increasing, may provide the more reliable localizing information.

The purpose of this study was to evaluate the feasibility of initial PET-CT in localization of the tumor bed through the method of deformable image registration with planning CT.

## Methods and materials

From November 2010 to July 2012, among breast cancer patients treated with breast conserving surgery and radiation therapy, we selected twenty-five patients who had a preoperative PET-CT performed and were implanted with more than two surgical clips in the tumor bed. This study was approved by the institutional review board of Ajou University Hospital. All patients were pathologically diagnosed with invasive ductal carcinoma except for one case of mucinous carcinoma and the age of patients ranged from 33 to 66 years (median, 46). Mean size of the primary tumor was 2.4 cm (range, 0.9-6.0) on diagnostic images. Diagnostic PET-CT was performed with the patients in the supine position and with both arms beside the trunk. Eight cycles of neoadjuvant chemotherapy was administered in eight patients (32%). After breast conserving surgery, the size of tumor ranged from 0.0 to 4.8 cm (mean, 1.8) on pathologic examination. Of 17 patients who did not receive neoadjuvant chemotherapy, 9 patients were treated with four to eight cycles of adjuvant chemotherapy.

**Figure 1:**
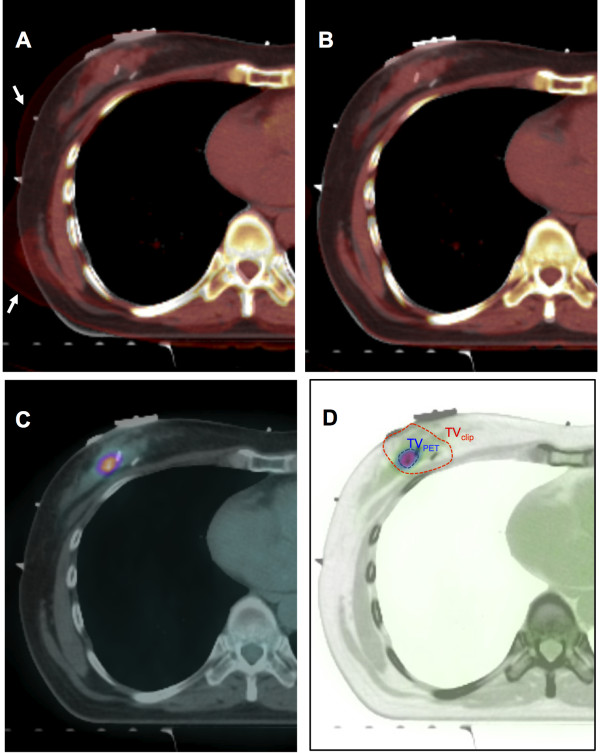
**Tumor bed localization procedure using deformable image registration of diagnostic PET-CT. A**. Automatic rigid image registration (DIR) process (arrows indicate the CT component of PET-CT), **B**. Deformable image registration (DIR) process between planning CT and CT component of PET-CT, **C**. Overlapped image between planning CT and PET component from PET-CT after DIR process, **D**. Target volume delineation of TV_PET_ and TV_clip._

For postoperative radiotherapy, non-enhanced planning CT was scanned with supine and ipsilateral arm-up position. Median interval between breast conserving surgery with/without adjuvant chemotherapy and planning CT was 4 weeks (range, 2–18). For deformable image registration (DIR) of PET-CT with planning CT, Velocity AI software version 2.7.0 (Velocity medical solutions, Atlanta, GA) was used (Figure 1A, 1B and 1C). Automatic rigid image registration (RIR) was done for the first step of DIR between diagnostic PET-CT and planning CT. After RIR, DIR was preceded to the next step. The spatial discrepancy of nipple between the two images was measured in each step and compared to evaluate the performance of the DIR process of breast tissue.

**Figure 2:**
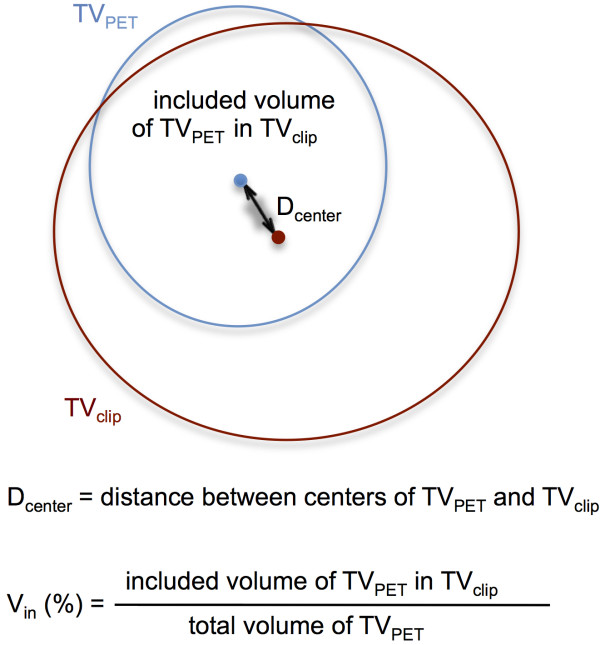
**Definition of D**_**center **_**(distance between centers of TV**_**PET **_**and TV**_**clip**_**) and V**_**in **_**(inclusion percent volume of TV**_**PET **_**included in TV**_**clip**_**).**

To investigate the feasibility of diagnostic PET-CT in localization of tumor bed, we delineated and compared two separate target volumes in each patient; 1) target volume based on surgical clips with a margin of 1 cm (TV_clip_) and 2) target volume based on 90% of maximum SUV on PET-CT registered by DIR (TV_PET_) (Figure 1D). Being blind to the PET-CT, each TV_clip_ was delineated by one radiation oncologist and reviewed and confirmed by two other radiation oncologists. TV_PET_ was delineated using the auto-segmentation tool of Velocity AI software referenced by the SUV. We analyzed the spatial relationship between TV_PET_ and TV_clip_ by the calculation of 1) D_center_: distance between center points of TV_clip_ and TV_PET_ and 2) V_in_ : inclusion percent volume of TV_PET_ included in TV_clip_ (Figure 2). All statistical analyses were performed with SPSS software, version 12.0 (SPSS, Inc., Chicago, IL, USA).

## Results

**Figure 3:**
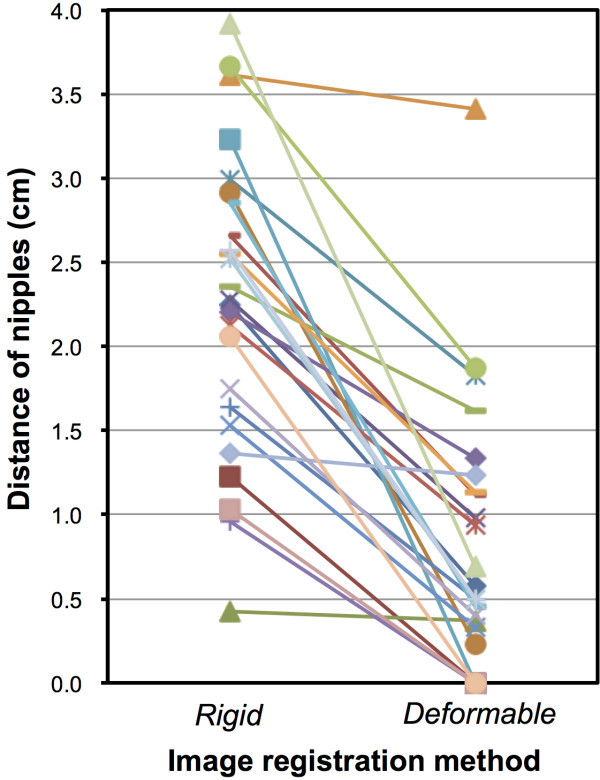
Change of distance of nipples between planning CT and PET-CT.

In the step of RIR, the distance between the nipples between PET-CT and planning CT ranged from 0.4 to 3.9 cm (mean, 2.3) and after the DIR process it ranged from 0.0 to 3.4 cm (mean, 0.8) (Figure 3). In 8 patients with no skin loss on the gross surgical specimen, the nipple distance ranged from 0.0 to 0.7 cm (mean 0.3 cm).

The median number of surgical clip was 3 (range, 2–6) and the volume of TV_clip_ ranged from 29.8 to 185.0 mL (mean 73.9, standard deviation 39.6). The median maximum SUV was 4.5 (range, 1.4-11.1) and the volume of TV_PET_ ranged from 4.7 to 122.1 mL (mean 49.1, standard deviation 33.85).

**Figure 4:**
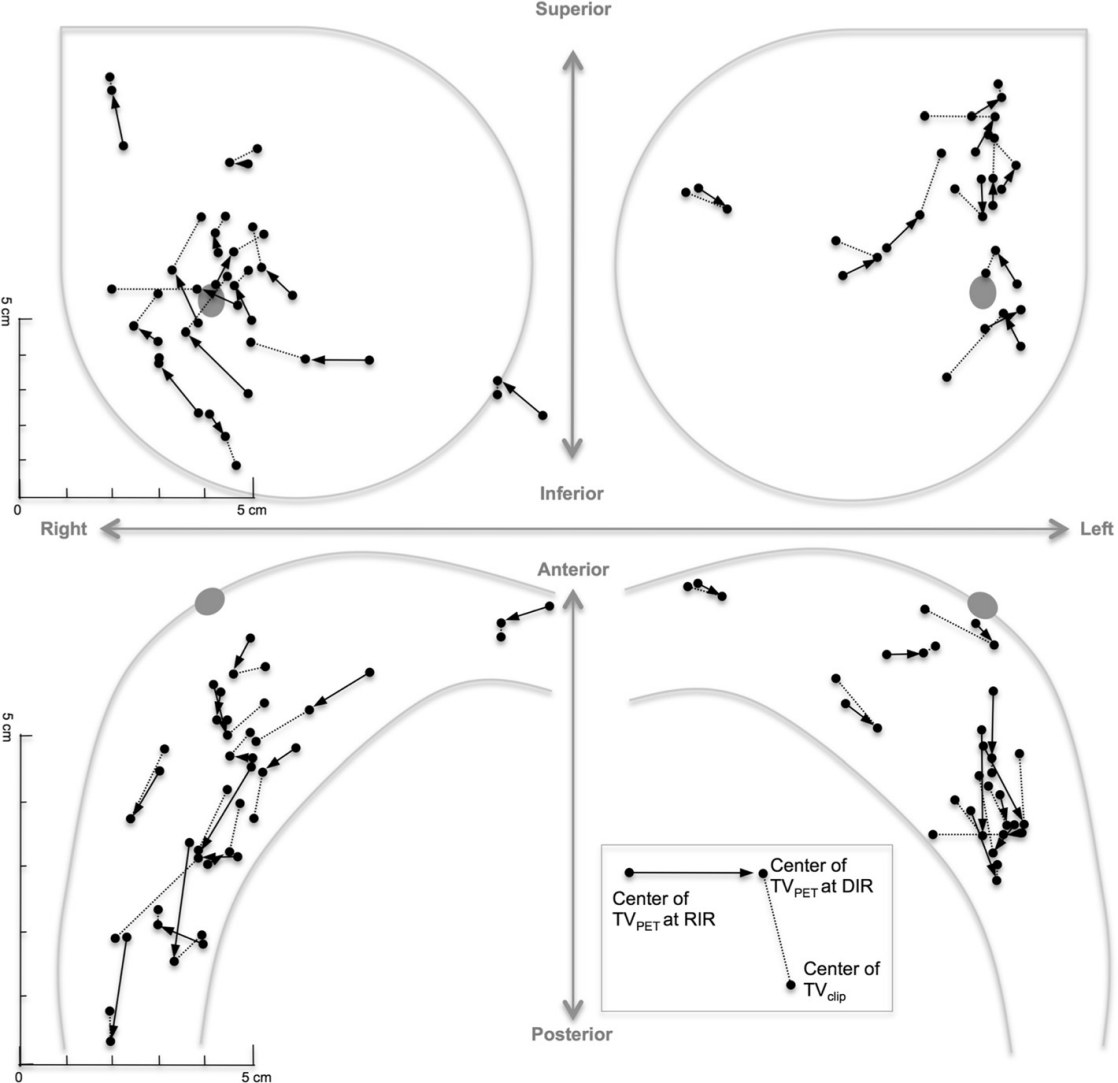
**Each patent’s geographic locations of centers of TV**_**clip**_**, TV**_**PET **_**at rigid image registration (RIR) step and TV**_**PET **_**at deformable image registration (RIR) step.**

Each patient’s geographic locations of center of TV_clip_, TV_PET_ at RIR and TV_PET_ at DIR were illustrated in Figure 4. The distance between TV_clip_ and TV_PET_ at DIR (D_center_) ranged from 0.3 to 2.5 cm (mean 1.4, standard deviation 0.6) and was statistically different from the distance between TV_clip_, TV_PET_ at RIR (mean 2.3, standard deviation 0.9) (paired t-test, p <0.01). When compared to the center of TV_clip_, the center of TV_PET_ at DIR tended to be located posteriorly (mean 0.3 cm, standard deviation 0.6), laterally (mean 0.3 cm, standard deviation 0.8) and inferiorly (mean 0.4 cm, standard deviation 0.9). The percent volume of TV_PET_ included in TV_clip_ (V_in_) ranged from 60.9 to 100% (mean 94.8, standard deviation 11.3) and 100% in 18 out of 25 patients.

## Discussion

In this study, we evaluated the feasibility of the DIR method using the diagnostic PET-CT to localize the tumor bed of breast cancer and the results demonstrate that the deformable registration of preoperative PET-CT can be a possible reference on the localization of tumor bed. The mean distance between centers of two target volumes (D_center_) was 1.4 cm and its maximum was 2.5 cm. For the purpose of direct localization and delineation of tumor bed based on TV_PET_, these levels of disagreement between two centers seem to be too high. However, the center of the TV_clip_ does not always represent the center of tumor volume because of the asymmetric surgical margin around the tumor. This asymmetry can affect the disagreement between the two centers of TV_clip_ and TV_PET_ in some degrees, and the D_center_ ranging from 0.3 to 2.5 cm can be an acceptable range to obtain additional information of tumor bed. Moreover, the high level of the inclusion percent volume of TV_PET_ in TV_clip_ (V_in_) suggests that the DIR method using PET-CT can be a possible tool in defining the tumor bed. Although the surgical clips/planning CT-based target volume delineation have been considered as the methods of choice for tumor bed radiotherapy, there may be some uncertainties associated with such methods. The surgical clips does not always represent the edge of tumor bed [7] and may migrate to the other site from its original location [13]. Lumpectomy-based delineation may yield a high discordance rate because of inter-observer variation [14]. Because of these uncertainties, multidisciplinary approach referenced by additional information should be considered for the accurate tumor bed boost. Recent studies have shown that the planning MR with planning CT helps in precise localization of tumor bed [6,8]. In these contexts, the localizing information obtained by the DIR method using preoperative PET-CT may possibly be one of the references for improving the accuracy of tumor bed boost.

The DIR method has been developed for the adopted radiation therapy, which allows more accurate irradiation considering the anatomic alteration caused by tumor regression or weight loss during the period of radiotherapy. In this study, we implemented DIR method to trace the tumor bed alteration due to tumor removal and the positional change. Because poor performance of the DIR method can make the tracing information unreliable, we checked the accuracy of DIR method by measuring the distance between the nipples between PET-CT and planning CT. Although the distance between the two nipples at the RIR step became closer after the DIR process (Figure 3), it was measured up to 3.4 cm, which is longer than what was expected. However, the excised skin after surgery can shift the nipple from its original site and in the patients with no skin loss on the gross specimen, the nipple distance was very close (range, 0.0 to 0.7 cm). This proximity between the nipples suggests that the TV_PET_ by the DIR process is reliably situated in the tumor bed. However, the accuracy of DIR algorithm should be tested in further studies, especially on the effects of tumor shrinkage or removal after chemotherapy or surgery.

This preliminary study has several shortcomings. The results are limited to the patients who had taken PET-CT as a preoperative work-up. The hypermetabolic lesions of PET-CT do not always exist and represent the true extent of breast tumor. Although we delineated the target volumes (TV_PET_ and TV_clip_) based on the relatively objective references such as the surgical clips and uptake value of PET, the intra or inter-observer variation may affect the validity of the results. In the evaluation of the image registration, nipple distance does not provide the sufficient data to ensure the appropriateness of the image matching and the standardized parameters representing the quality of the match should be developed in the breast tissue. The surgical removal of breast tissue, tumor regression after neoadjuvant chemotherapy and long time interval between surgery and radiotherapy may influence the accuracy of tumor bed localization. Therefore, it is proposed that the results of this study should be applied with caution as supplementary information adding to the standard references. Nonetheless, our results may provide a reference in the case of patients who have no other reliable references. The DIR method can also be a useful tool for better definition of tumor bed in the patients treated with neoadjuvant chemotherapy or the oncoplastic surgery [15].

## Conclusion

In conclusion, using the DIR method, initial diagnostic PET-CT can be one of the possible references to localize the tumor bed in breast cancer radiotherapy.

## Abbreviations

DIR: Deformable image registration; RIR: Rigid image registration; TV: Target volume.

## Competing interests

The authors declare no conflict of interest.

## Authors’ contributions

OKN, OC and MC designed the research, OKN and OC performed the research, OC, HP, MK and JH analyzed the data, OKN, OC and YO wrote paper. All authors read and approved the final manuscript.
